# Phytochemical and Micro-Morphological Characterization of *Atraphaxis pyrifolia* Bunge Growing in the Republic of Kazakhstan

**DOI:** 10.3390/molecules29040833

**Published:** 2024-02-13

**Authors:** Alima Abilkassymova, Raushan Kozykeyeva, Jennyfer Andrea Aldana-Mejía, Sebastian John Adams, Ubaidilla Datkhayev, Aknur Turgumbayeva, Kulpan Orynbassarova, Seethapathy G. Saroja, Ikhlas A. Khan, Samir A. Ross

**Affiliations:** 1 Higher School of Medicine, Al-Farabi Kazakh National University, Almaty 050040, Kazakhstan; abilkasymova_a@mail.ru (A.A.); turgumbayeva.aknur@med-kaznu.com (A.T.); 2 School of Pharmacy, Asfendiyarov Kazakh National Medical University, Almaty 050012, Kazakhstan; u.datkhayev@gmail.com; 3 National Center for Natural Products Research, School of Pharmacy, The University of Mississippi, Oxford, MS 38677, USA; nar_rau@mail.ru (R.K.); jaaldana@olemiss.edu (J.A.A.-M.); jasabest@olemiss.edu (S.J.A.); seethapathy.gs@gmail.com (S.G.S.); ikhan@olemiss.edu (I.A.K.); 4 Department of Pharmacognosy, Faculty of Pharmacy, South Kazakhstan Medical Academy, Shymkent 160019, Kazakhstan; kulpan_ok@mail.ru; 5 School of Life Sciences, University of Westminster, London W1W 6UW, UK; 6 Department of Biomolecular Sciences, Division of Pharmacognosy, School of Pharmacy, University of Mississippi, Oxford, MS 38677, USA

**Keywords:** *Atraphaxis pyrifolia*, flavonoids, triterpenes, micro-morphology, HPTLC

## Abstract

*Atraphaxis pyrifolia* is a native species of Central Asia, known for curing several disorders. The species has little knowledges about its chemical composition and any information about its morphological characteristics despite its importance in traditional Asian medicine. This is one of the first approaches to the phytochemical and morphological characterization of this species. Micro-morphology was performed on the stem, and leaf parts of this plant to profile the morpho-anatomical characters using brightfield, fluorescence, polarized and scanning electron microscopy. Leaves were extracted with hexane and methanol. The hexane extract was analyzed using GC-MS analysis revealing the major presence of γ-sitosterol and nonacosane. The methanolic extract was submitted to Vacuum Liquid Chromatography and Sephadex LH-20. HPTLC, HR-ESI-MS and NMR techniques were used to identify the main compounds. Four glycosylated flavonoids were isolated: 8-*O*-acetyl-7-*O*-methyl-3-*O*-α-l-rhamnopyranosylgossypetin (Compound **1**), and 7-*O*-methyl-3-*O*-α-l-rhamnopyranosylgossypetin (Compound **3**), and two other compounds reported for the first time in the literature (Compounds **2** and **4**). The findings presented herein furnish pertinent information essential for the identification and authentication of this medicinal plant. Such insights are invaluable for facilitating robust quality control measures and serve as a foundational framework for subsequent endeavours in metabolic, pharmacological, and taxonomical analyses.

## 1. Introduction

The family Polygonaceae consists of herbs, shrubs and small trees, characterized by simple leaves with sheaths. It includes about 59 genera covering a total of 1384 species [1]. The genus *Atraphaxis* L. comprises approximately 35 species distributed throughout Northeast Africa and Eurasia, from Southeast Europe to Eastern Siberia, China and Mongolia [2,3,4]. It is widely represented in the territories of Kazakhstan on rocky and gravelly slopes of mountains in in environments characterized by saline, gravelly, and stony substrates, as thickets of shrubs or subshrubs [5].

In Turkmenistan’s folk medicine, the powder of *A. pyrifolia* shoots is used for gastroenteritis [6]. According to Uzbekistan traditional knowledge, *A. pyrifolia* leaf infusion enhance cardiac function and promotes blood circulation, alleviating headaches, insomnia, and tinnitus, while also bolstering overall bodily vigor [7]. Currently, there are no available reports on the pharmacological evaluation of *A. pyrifolia*. However, the *Atraphaxis* genus has shown promising pharmacological properties such as antioxidants, antimicrobial effects, and therapeutic benefits. Furthermore, it has been used to treat diseases of the lymphatic system, bacterial fevers, throat infections, and even eye diseases like cataracts [8], properties that can be related to its chemical composition.

Earlier phytochemical studies on the genus *Atraphaxis* have shown that this genus is rich in various types of chemical components, including phenylpropanoids, flavonoids, anthraquinones, sterols, benzenoids, tannins and phenolic compounds [9]. Flavonoid glycosides have been found to be the major components of *Atraphaxis* species [10]. Few early studies on the phytochemistry of *A. pyrifolia* leaves reported the compounds: 7-*O*-methylgossypetin-3-*O*-α-l-rhamnopyranoside [11], 7-*O*-methylluteolin 4’-*O*-*β*-d-glucofuranosyl-(1→6)-d-glucopyranoside [12]; 8-acetylmethylgossypetin 3-*O*-α-l-rhamnopyranoside (pyrifolin), its aglycone 8-acetyl-7-methylgossypetin (pyrifolidin), and 7-methylgossipetin 3-*O*-α-l-rhamnopyranoside (pyrifolinin) [13]; 7-methylgossypetin 8-*β*-d-glucopyranoside 3-*O*-α-l-rhamnopyranoside and 7-methylgossipetin 8-*β*-d-glucopyranoside [14].

Detailed micro-morphology studies of the shoots, leaf blades, and pollen on the genus *Atraphaxis*, have been conducted [15], and more recently from the exocarp [5], for the correct classification of this genus taxonomically. The habit of the genus *Atraphaxis is* dwarf or shrubs of 30–150 cm tall, with elongated leaves and short axillary branchlets, characteristic of this genus [4]. However, no studies have been carried out on the leaf and stem anatomy of *A. pyrifolia*.

This research aimed to profile the plant species using macroscopy, and phytochemical analysis using different chromatographic techniques. A comprehensive characterization of this species, in addition to contributing to its understanding, will also enable the exploration of the correlation between its traditional use and its chemical profile. Furthermore, it will establish the foundations for future analysis and validation of its botanical samples.

## 2. Results

### 2.1. Identification of Secondary Metabolites in the Hexane Extract from the Leaves of A. pyrifolia Using GC-MS

By using GC-MS, 12 major compounds from the hexane extract of the leaf were identified in *A. pyrifolia* by comparing their mass spectra with the NIST Library (version 2.3, 2017). The identified compounds, their retention indices, and their percentage compositions were summarized in Table 1.

### 2.2. Extraction and Isolation of Secondary Metabolites from the Methanolic Extract from the Leaves of A. pyrifolia

**Figure 1:**
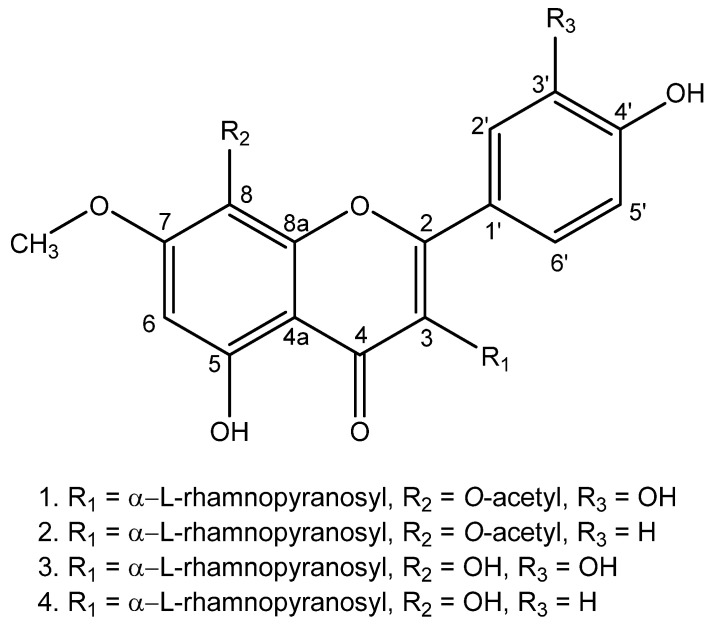
Structure of isolated compounds **1**–**4**.

By using the VLC technique, fractionation of methanolic extract of *A. pyrifolia* leaves was obtained using the gradient elution of DCM: MeOH, resulting in an enriched fraction of flavonols. The isolation process via the Sephadex LH-20 gives rise to the isolation of four flavonols (Figure 1). Two of the isolated substances are the previously reported compounds 8-*O*-acetyl-7-*O*-methyl-3-*O*-α-l-rhamnopyranosylgossypetin (Compound **1**) and 7-*O*-methyl-3-*O*-α-l-rhamnopyranosylgossypetin (Compound **3**), the other two compounds are reported for the first time: 5-hydroxy-2-(4-hydroxyphenyl)-7-methoxy-4-oxo-3-(((3*S*,4*S*,6*S*)-3,4,5-trihydroxy-6-methyltetrahydro-2*H*-pyran-2-yl)oxy)-4*H*-chromen-8-yl acetate (Compound **2**), 5,8-dihydroxy-2-(4-hydroxyphenyl)-7-methoxy-3-(((3*S*,4*S*,6*S*)-3,4,5-trihydroxy-6-methyltetrahydro-2*H*-pyran-2-yl)oxy)-4*H*-chromen-4-one (Compound **4**) (Figure 1, Table 2).

### 2.3. High-Performance Thin-Layer Chromatography

**Figure 2:**
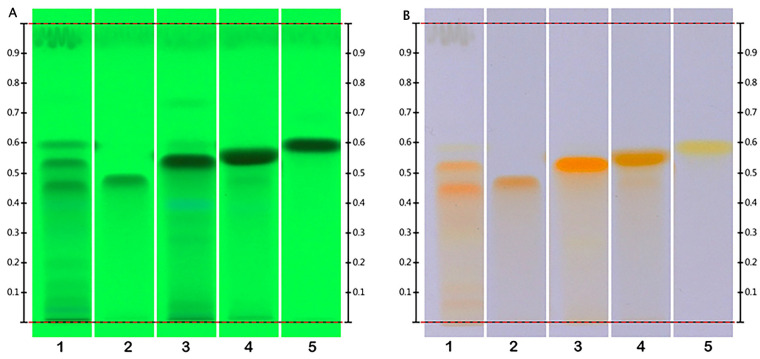
HPTLC fingerprints of leaf extract. (**A**). Under 254 nm before derivatization: (**B**). Under white light after derivatization using natural products reagent. Lane 1—methanol extract of *A. pyrifolia* leaves, lane 2—Compound **3**, lane 3—Compound **1**, lane 4—Compound **4**, lane 5—Compound **2**.

In the present work, the HPTLC fingerprint profile was developed for the leaves crude extract of *A. pyrifolia* and the four isolated flavanols (Figure 2). Furthermore, the compounds at the Rf 0.47, 0.53, 0.55 and 0.59 were confirmed as flavanols using the Naturstoff reagent A (diphenylboric acid 2-aminoethyl ester [DPBA]) derivatives reagent (Figure 2).

### 2.4. Macro and Microscopic Description of Leaf and Stem of A. pyrifolia

The external morphology of the stem is tortuous, with gray-brown hard barks; outer epi-dermal covers split and the inner wood is grayish white. Leaves are green, broadly elliptic or obovate, 1.5–2.5 × 1–1.3 cm, both surfaces glabrous, and the venation is very prominent in the abaxial surface, the leaf margin is entire to slight crenate.

**Figure 3:**
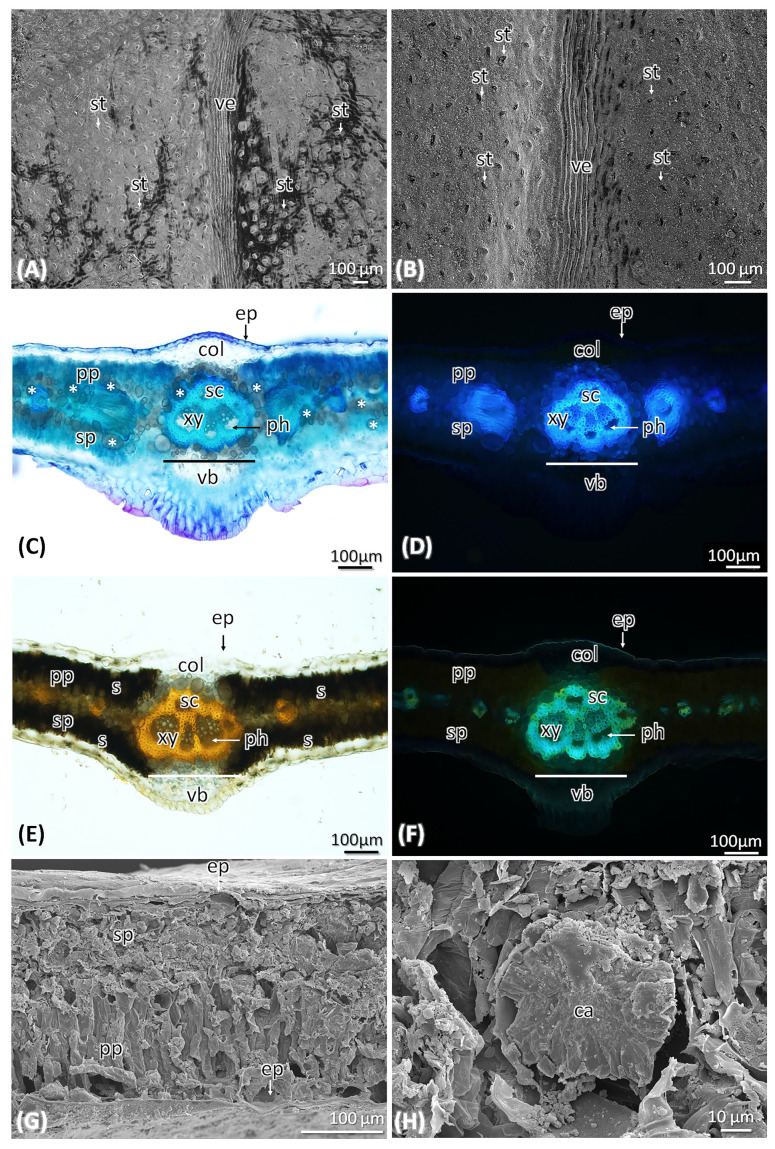
Surface view and transverse section of leaf of *A. pyrifolia*. (**A**,**B**). Ultra-structural surface view of leaf using Scanning Electron Microscopy (SEM). (**A**). Leaf upper surface, (**B**). Leaf lower surface, both showing the presence of stomata (st). (**C**). Leaf section observed under visible light, stained with Toluidine blue O. Calcium oxalate crystals marked with (*). (**D**). observed under UV, auto- fluorescence of lignified cells in the leaf. (**E**). Presence of starch grains in the palisade parenchyma cells of the leaf, stained with Lugol’s iodine. (**F**). Stained for cutin and lignin with Auramine O. (**G**). Transverse section of leaf showing the internal structure, observed under SEM. (**H**). Close view of calcium oxalate crystal present in the leaf. ep—epidermis, col—collenchyma cells, pp—palisade parenchyma cells, sc—sclerenchyma cells, xy—xylem, ph—phloem, sp—spongy parenchyma cells, vb—vascular bundles, ve—vein, s—starch grains, st—stomata, ca—calcium oxalate crystals.

The isobilateral leaf is covered with a thin cuticular layer on both surfaces. The leaf is amphistomatic, having stomata on both surfaces; these stomata are at the same layer of epidermis cells (Figure 3A,B). *A. pyrifolia* leaf epidermis is a single layer with oval to circular shaped cells. Mesophyll cells consist of 2–3 layers of palisade parenchyma tissue and are present on the adaxial and abaxial sides of the leaf blade. In between these two palisade parenchyma layers, the vascular strands pass through the lamina (Figure 3C–G).

The abundance of druses/spherical calcium oxalate crystals present in the mesophyll cells, which are larger in diameter of 140–160 µm (Figure 3H). Chlorenchyma is differentiated into palisade parenchyma and shows the presence of secondary metabolites and an abundance presence of starch grains (Figure 3E). Vascular bundles of different sizes are arranged in a line located in the middle region of the mesophyll. The grouped vascular bundle is a board in the midrib and consists of four vascular bundles. Three vascular bundles show the phloem towards the abaxial and xylem towards the adaxial side and in one bundle it is the opposite. Lateral vascular bundles are also evident in the leaf lamina (Figure 3C–F). Sclerified cells are located around the vascular bundles along with the phloem and xylem. There are absences of secretory structures and subepidermal cavities in the leaf blades.

**Figure 4:**
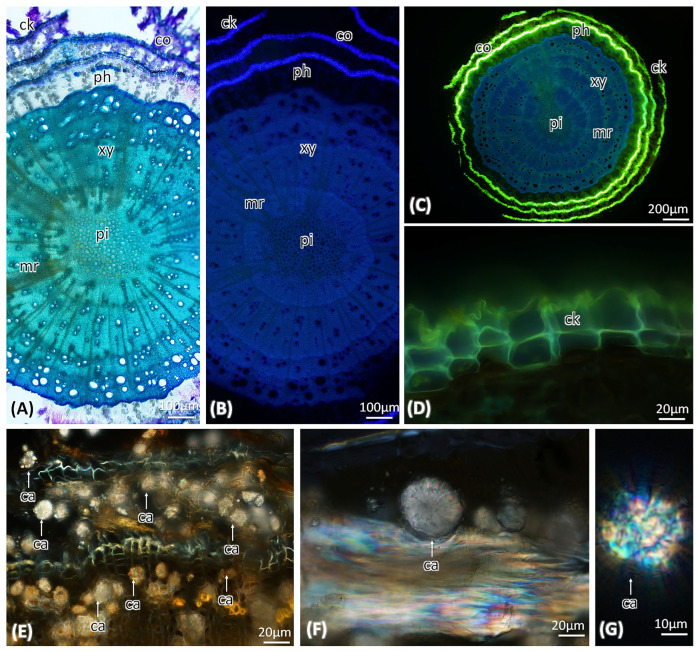
Transverse section of stem. (**A**). Toluidine Blue O stained section viewed under visible light. (**B**). Same was observed under UV. (**C**,**D**). Stained with Fluorol Yellow 088, it shows the strong UV active cork-cortex cells. (**E–G**). presence of calcium oxalate crystals, visible clearly under polarized light. ck—cork, co—cortex, ph—phloem, xy—xylem, mr—medullary rays, pi—pith, ca—calcium oxalate crystals.

The transverse section of the stem is composed of several layers of lignified cork. Followed by the phloem patches composed of phloem sieve tube elements and companion cells. The presence of calcium oxalate crystals in the phloem rays and parenchyma cells was observed. The wood has distinct growth rings. Wood with diffuse or semi-ring porous, vessels, 2–4 cells in clusters and some are solitary. Medullary rays are biseriate and rarely multiseriate. The central region is composed of parenchymatous pith (Figure 4A–G).

## 3. Discussion

The steroid *γ*-sitosterol emerged as the principal compound in the hexane extract of *A. pyrifolia* leaves. According to the National Center for Advancing Translational Sciences [16], this natural compound shows potential for treatment for diabetes in rodents; however, caution is advised regarding its use as a natural supplement due to its observed toxicity in in-vitro human cell assays. Additionally, alkanes such as nonacosane and triterpenes as lup-20(29)-en-3-one were identified as major compounds in the sample. Lupeol, has been demonstrated to function as an immunomodulator, displaying biological properties including anti-inflammatory, anti-arthritis, antioxidant and anticancer effects [17]. 

The methanolic extract of *A. pyrifolia* leaves was submitted to different chromatographic techniques, reaching the isolation of 4 flavonoid compounds. The isolated compounds were characterized by their structural elucidation via 1D (^1^H, ^13^C) and 2D NMR (HSQS, HMBC) and confirmed using HR-ESI-MS. Compound **1** was previously reported on *A. frutescens* aerial parts [8], but this is the first report of its isolation in the leaves of *A. pyrifolia*. Compound **3** was formerly isolated from *A. pyrifolia* leaves [11] and is also present on *A. frutescens* aerial parts [8]. The ^1^H and ^13^C NMR data and HR-ESI-MS data (See Supplementary Material) agreed with the previously published data. On the ^1^H spectrum, all compounds shared features of an aromatic A-ring proton singlet (δH 6.46–6.51). In the ^13^C NMR spectrum, a carbon resonance at δC 158.72–159.63 of C-2, and a carbonyl resonance at δC 179.59–180.12 of C-4, indicated a flavonol moiety. A doublet with δH 5.36–5.39 from the anomeric proton, correlated to the C-3 (δC 135.90–136.39), evidences glycosylation on this position. The presence of an aliphatic carbon at δC 17.56–17.66 with a corresponding methyl doublet resonance in the HSQC at δH 0.93–0.95 (d, 3H, Rha-6) suggested the presence of a rhamnopyranosyl moiety.

Additionally, compounds **1** and **2** presented two singlets in the NMR spectra. The first one corresponds to a methoxy singlet δH 3.87–3.89 (for 3H each), correlated to a C-7 (δC 158.63–160.4) in the HMBC spectra. The second singlet corresponds to a methyl proton resonance at δH 2.28–2.33, correlated to a carbonyl carbon resonance at δC 170.26–170.36 using the HMBC analysis, indicating an acetyl group at C-8. In compounds **3** and **4**, a δC of 127.53–127.56 of C-8 indicated the absence of the carboxylic group at this position.

This is the first report of the presence of compound **2** in *A. pyrifolia*. The molecular formula of this compound was established as C_24_H_24_O_12_ based on the HR-ESI-MS data which showed a molecular ion peak [M + H]^+^ at *m*/*z* 505.137 (calcd. C_24_H_24_O_12_, 505.13455). The ^1^H NMR spectrum showed the main features of a flavonoid, as described for compounds **1** and **3**, with a rhamnopyranosyl moiety at C-3. However, for this compound the NMR data showed a phenol B-ring instead of a catechol ring, confirmed using a hydroxylated carbon resonance at δC 161.79 of C-4′, and aromatic protons duplets (δH 7.69 d, *J* = 8.9 Hz, 2H, H-2′/H-6′; δH 6.93, d, *J* = 8.9 Hz, 2H, H-3′/H-5′). The HMBC analysis indicated a correlation between the protons H-2′/H-6′ and H-3′/H-5′, with the δC 161.79 of C-4′, while the H-2′/H-6′ were long-range coupled with δC 159.12 corresponding to the C-2 position.

Additionally, compound **4** is being reported for the first time. The molecular formula of this compound was established as C_22_H_22_O_11_ based on the HR-ESI-MS data which showed a molecular ion peak [M − H]^−^ at *m*/*z* 461.105 (calcd. C_22_H_22_O_11_, 462.11616). As well as compound **2**, NMR data showed a phenol B-ring instead of a catechol ring, confirmed using a hydroxylated carbon resonance at δC 161.58 of C-4′, and aromatic protons multiplets at δH 7.87, 2H, H-2′/H-6′ and δH 6.94, 2H, H-3′/H-5′. The HMBC analysis indicated a correlation between the protons H-2′/H-6′ and H-3′/H-5′, with the δC 161.58 of C-4′, while the H-2′/H-6′ were long-range coupled with δC 159.63 corresponding to the C-2 position.

In the study conducted by Odonbayar et al. [8], flavonol glycosides demonstrated significant tyrosinase inhibition in mushroom and antioxidant activities. The research suggests that the presence of the 3-*O*-rhamnopyranosile moiety in flavonols could play a crucial role in inhibiting the tyrosinase enzyme.

In this study, an analysis of the methanolic extract from the leaves was conducted by HPTLC to assess its qualitative chromatographic profile. HPTLC, an automated and efficient progression from Thin Layer Chromatography (TLC), is employed for a comprehensive quality assessment and evaluation of plants fingerprints [18]. The acquisition of these chemical profiles serves as a valuable tool for the evaluation of botanical materials from *A. pyrifolia* and even from the genus *Atraphaxis*, with a focus on flavonol glycosides as key chemical markers.

Regarding anatomical studies *A. pyrifolia*, detailed information is lacking. Some morpho-anatomical comparisons of this genus have been made in the past. External morphology characters such as highly lignified, prickly elongated shoots and branchlets [4], thick leaves, and leathery leaf blades, were used to distinguish this species from other species of this genus [19]. The micromorphology of the inflorescences and pollen of *A. pyrifolia* was well studied [19], but the anatomy of the stem and leaf are not highly mentioned. The wood anatomy of several *Atraphaxis* species, including *A. billardieri*, *A. billardieri* subsp. *tournefortii*, *A. spinosa*, and *A. grandiflora*, have been studied. However, studies on the stem of *A. pyrifolia* are lacking [20]. Our study found that the wood of *A. pyrifolia* is diffuse to semi-ring porous, like *A. grandiflora*. The medullary rays are 1–2 cells thick, like *A. billardieri*, *A. billardieri* subsp. *tournefortii*, *A. grandifolia*, and *A. spinosa*. The presence of multiseriate rays is highlighted in the family Polygonaceae; however the genus *Atraphaxis* stem consists of 1–2 cell thick ray cells [20]. The presence of calcium oxalate crystals, which is large in both the leaf and stem, is a characteristic feature. 

The chemical characterization of *A. pyrifolia*, a species insufficiently explored in the literature, along with the evaluation of its TLC profiles—previously unestablished for this plant—and its macro-microscopic characterization, aids in the botanical authentication and identification, contributing to a more comprehensive understanding of the genus.

## 4. Materials and Methods

### 4.1. General Experimental Procedures

TLC was performed on pre-coated SiO_2_ 60 F_254_ TLC plates (0.2 mm). The detection of the compounds used UV absorption (λ_max_ 254 and 366 nm) and for derivatization anisaldehyde, and Naturstoff reagents. HPTLC was performed using CAMAG automatic TLC sampler-4. Derivatization was performed in the CAMAG chromatogram immersion de-vice-III using the mobile phase EtOAc: DCM: MeOH: DW (15:8:4:1) *v*/*v*. Agilent 7890A gas chromatographic (GC) instrument, equipped with an Agilent 7693 autosampler and connected to an Agilent 5975C mass spectrometer (Agilent, Santa Clara, CA, USA), was used to conduct GC-MS. NMR analyses were performed on Bruker mode AMX 500 and AMX 400 NMR spectrometers (Bruker, Billerica, MA, USA); a standard pulse system collected ^1^H and ^13^C NMR spectra. The DEPT and 2D-NMR experiment (HMBC) was carried out using standard Bruker pulse programs. The instrument ran at 500 MHz and 400 MHz in ^1^H and 125 MHz in ^13^C. Deuterated solvent (99.8% purity, Sigma-Aldrich, Darmstadt, Germany) methanol (CD_3_OD) was used for NMR analysis. The liquid chromatographic system employed in this study was an Agilent Series 1200 HPLC system (Agilent Santa Clara, CA, USA) equipped with a Quaternary pump and an Autosampler, hyphenated to a ToF-MS (Model #G6230B) from Agilent Technologies equipped with an ESI source. All operations, data acquisition, and analysis were controlled using Agilent MassHunter Acquisition Software Ver. A.10.1 and was processed using MassHunter Qualitative Analysis software Ver. B.10.00. HPLC-grade water and acetonitrile, with 99.9% of purity were purchased from Fisher Scientific for the HR-ESI-MS analysis. For other chromatographic processes, 99.8% of purity laboratory grade dichloromethane (DCM), ethyl acetate (EtOAc), methanol (MeOH), and deionized water (DW) were purchased from Fisher Scientific (Thermo Fishcer Scientific, Fair Lawn, NJ, USA).

### 4.2. Plant Material

The aerial parts of *A. pyrifolia* plant were collected in May 2023 from the Karakus Mountains near Sastobe village in the Turkestan region of Kazakhstan (42°31′45.00″ N, 70°06′20.0″ E). Steams were separated from the leaves, and the plant materials were dried at room temperature 25 °C. Identification and authentication of the plant was performed by Dr. Mikhail Danilov, Institute of Botany and Phytointroduction, Almaty, Kazakhstan. The collected plant was stored in the Al-Farabi KazNU, Almaty, Kazakhstan with voucher numbers 01-05/518. The plant materials were assigned unique NCNPR numbers # 25799 (stem) and 25780 (Leaf) and stored at the Botanical Repository of the National Center for Natural Products Research, University of Mississippi, USA.

### 4.3. Identification of Secondary Metabolites in Hexane Extract of A. pyrifolia Leaves by GC-MS

Air-dried powdered leaves (450 g) of *A. pyrifolia* were extracted with 2000 mL of hexane overnight and then submitted to ultrasound extraction for 1 h. This procedure was repeated three times with 30 min intervals over 3 days. Then, the extract was filtered, and the solvent evaporated to yield 11 g (2.44%).

Analysis of the hexane extract was performed utilizing an Agilent 7890A gas chromatographer (GC) with an Agilent 7693 autosampler and an Agilent 5975C mass spectrometer (Agilent, Santa Clara, CA, USA). The capillary column (60 m × 0.25 mm i.d.) utilized was coated with a 0.25 µm thick film of 100% Dimethylpolysiloxane (Agilent DB-1MS). Helium at a constant flow rate of 1 mL/min was used as the carrier gas. Each sample was analyzed using the following GC oven program: 50 °C held for 2 min, then heated at a rate of 5 °C/min to 280 °C and held at 280 °C for 15 min. The inlet was programmed to 280 °C in split mode, with a split ratio of 25:1. The sample injection volume was 1 µL.

The Agilent 5975C mass spectrometer was operated with an electron energy of 70 eV. The source, quadrupole, and transfer line temperatures were 230 °C, 150 °C, and 280 °C, respectively, during the experiment. All mass spectra data were recorded from 40 to 450 *m*/*z* after a 10.5 min solvent delay. The NIST Mass Spectral Library (NIST 17) and NIST Mass Spectral Search Program (Version 2.3), were used for tentative compound identification.

### 4.4. Extraction and Isolation of Secondary Compounds from Leaves Methanolic Extract

Dried *A. pyrifolia* leaves were grounded and submitted to ultrasonic extraction, using an ultrasonic Bransonic 5800 series (Branson, Brookfield, CT, USA). A total of 400 g of dried *A. pyrifolia* leaves were extracted with 1600 mL of methanol at 40 °C for 1 h. The extract was filtered, and the residue was resubmitted to extraction, repeating the process five times at 30 min intervals over 3 days. The methanolic extract was evaporated using a rotary vacuum evaporator until dried, affording 42.26 g crude methanolic extract. Then, 4.8 g of methanolic extract was mixed with 10 g Celite and fractionated on 200 g silica column using vacuum liquid chromatography eluted with DCM: MeOH mixtures of increasing polarities starting with 100% DCM then MeOH was increased gradually (100, 98:2, 96:4, 94:6, 92:8, 90:10 and 100). The resulting fractions were screened on silica gel TLC sprayed with vanillin-sulphuric acid, anisaldehyde, and DPBA reagent spraying agents. Nine fractions were obtained (A to I). Fraction H (640 mg) was subjected to further separation on Sephadex LH-20 (30 g) column chromatography eluted with 100% MeOH-affording compounds: **1** (7.5 mg), **2** (15.8 mg), and **3** (66.9 mg). Fraction G and subfractions 42–50 from H combined (319.3 mg) and subjected to further separation on Sephadex LH-20 (30 g) column chromatography eluted with 100% MeOH affording compound **4** (9.4 mg).

**Compound 1** (8-*O*-acetyl-7-*O*-methyl-3-*O*-α-l-rhamnopyranosylgossypetin): yellow powder. NMR see Table 2, data as reported by Odonbayar [8] C_24_H_24_O_13_ based on the HR-ESI-MS data (*m*/*z* 521.147 [M + H]^+^; calcd. for C_24_H_24_O_13_, 521.12892).

**Compound 2** (5-hydroxy-2-(4-hydroxyphenyl)-7-methoxy-4-oxo-3-(((3*S*,4*S*,6*S*)-3,4,5-trihydroxy-6-methyltetrahydro-2*H*-pyran-2-yl)oxy)-4*H*-chromen-8-yl acetate): yellow powder. NMR data see Table 2. C_24_H_24_O_12_ based on the HR-ESI-MS data (*m*/*z* 505.137 [M + H]^+^; calcd for C_24_H_24_O_12_, 505.13455).

**Compound 3** (7-*O*-methyl-3-*O*-α-l-rhamnopyranosylgossypetin): yellow powder. For NMR see Table 2, data as reported by Odonbayar [8]. C_22_H_22_O_12_ based on the HR-ESI-MS data (*m*/*z* 477.099 [M − H]^−^; calcd for C_22_H_21_O_12_, 477.10323).

**Compound 4** (5,8-dihydroxy-2-(4-hydroxyphenyl)-7-methoxy-3-(((3*S*,4*S*,6*S*)-3,4,5-trihydroxy-6-methyltetrahydro-2*H*-pyran-2-yl)oxy)-4*H*-chromen-4-one): yellow powder. NMR see Table 2. C_22_H_22_O_11_ based on the HR-ESI-MS data (*m*/*z* 461.105 [M − H]^−^; calcd for C_22_H_21_O_12_, 462.11616).

The sugar moiety of the compounds and their arrangement were identified following the method reported by Mohamed et al. [21]. Compounds were hydrolyzed using 2 M HCl at 90 °C for 2 h. Post-hydrolysis, the reaction mixture was neutralized with 200 μL of 9 M NH_4_OH and dried using N_2_ gas. The hydrolysis products were derivatized and analyzed using UHPLC-UV-MS, and then their retention time and mass spectra were compared with those of derivatized monosaccharide standards.

### 4.5. HR-ESI-MS Analysis

The isolated compounds were analyzed on a liquid chromatographic system using a mobile phase composed of water with 0.1% formic acid (A) and acetonitrile with 0.1% formic acid (B) at a flow rate of 0.3 mL/min. The analysis was conducted on a 150 mm × 3.0 mm, 5 μm Luna C18(2) column (Phenomenex, Torrance, CA, USA), with a gradient of 10% A and 90% B, reaching 100% of B in 30 min. Temperature was adjusted at 40 °C. A sample volume of often microliters was injected.

Mass spectrometric analysis was carried out using a ToF-MS with an ESI source. The analysis employed the following parameters: drying gas (N_2_) flow rate of 7 L/min, drying gas temperature set at 300 °C, nebulizer pressure of 25 psig, sheath gas temperature at 300 °C, sheath gas flow of 7 L/min, capillary voltage set to 3 kV, nozzle voltage at 1 kV, skimmer set at 65 V, Oct RF V at 750 V, and fragmentor voltage at 120 V. For the mass analysis, each sample was examined in positive and negative mode within the range of *m*/*z* = 200–900, utilizing an extended dynamic range (flight time to *m*/*z* 1700 at a 2 GHz acquisition rate).

### 4.6. High-Performance Thin Layer Chromatography

Approximately 1.5 mg of the dry crude leaf extract of *A. pyrifolia* and the isolated compounds were suspended in 1 mL of methanol. Each sample was filtered using the Samplicity filtration system prior to the HPTLC analysis. Chromatography was performed on HPTLC silica plates (10 × 10 cm) pre-coated with silica gel 60 F_254_, applying 8 mm wide bands with a Camag automatic TLC sampler-4 positioned 8 mm from the lower edge of the plate at an application rate of 100 nL/sec with an application volume of 10 µL. The plates were developed using a chromatography chamber with a mobile phase containing ethyl acetate: dichloromethane: methanol: water (15:8:4:1). The development chamber was first saturated by the mobile phase with a saturation pad for 20 min at room temperature (21 ± 1 °C). The plate was activated at a relative humidity of 35 ± 5%. The development was stopped when the solvent frontline reached 70 mm from the plate’s lower edge. Derivatization was performed in the Camag chromatogram immersion device-III with a dipping speed of five and a dipping time of one second using a derivatizing agent containing DPBA to confirm the presence of flavonoids. After immersion, the plates were heated at 110 °C for 10 min in a Camag TLC pate heater-III. The plates were examined under 254 nm prior to derivatization and examined under white light after derivatization using the Camag TLC visualizer. Rf values and images were recorded using VisionCATS software (Version 2.1).

### 4.7. Macro and Microscopic Description of Stem and Leaf of A. pyrifolia

In detailed anatomical studies, several sections of leaf, and stem were hand-sectioned (~50 µm thick). For uniformity, the middle part of each leaf was sectioned and stained with Toluidine Blue O (TBO) for basic histology observation. Lugol’s iodine was used for starch localization [22]. Auramine O was used for lignin and cutin, and fluoral yellow 088 was used for total lipid compounds [23,24]. All mounts were prepared on glass slides with water. Photomicrographs were obtained using an Olympus BX53 compound microscope equipped an Olympus DP74 camera system (Olympus, Shinjuku, Tokyo, Japan) with fluorescence imaging. Images were processed using OLYMPUS CellSens standard 2 (version 3.1, build 21199) imaging software (Olympus Corp., Tokyo, Japan).

#### Preparation of Samples for Scanning Electron Microscopy (SEM)

Specimens fixed in FAA [Formaldehyde (10%): Alcohol (50%): Acetic Acid (5%)] in 35% of deionized (DI) water. Before starting the dehydration process, the samples were washed with water and passed through 30%, 50%, 70%, 90%, and 100% ethanol solutions. The samples were dehydrated using a Leica CPD300 critical point dryer (Leica Microsystems, Wetzlar, Germany) supplied with liquid CO_2_; dried samples were mounted on aluminum stubs with double-sided adhesive carbon tape then coated with platinum using a Desk V HP sputter coater (Denton Vacuum, Moorestown, NJ, USA) supplied with argon gas. The samples were imaged using a JSM-7200FLV field-emission SEM (JEOL Ltd., Tokyo, Japan).

## 5. Conclusions

The GC-MS characterization of hexane extract from *A. pyrifolia* leaves identify the γ-sitosterol and lup-20(29)-en-3-one, and the alkane nonacosane, as major non-polar compounds. Additionally, the methanolic extract of the leaves yielded four flavonol glycosides, two of them being reported for the first time. Specifically, Compound **2** (5-hydroxy-2-(4-hydroxyphenyl)-7-methoxy-4-oxo-3-(((3*S*,4*S*,6*S*)-3,4,5-trihydroxy-6-methyltetrahydro-2*H*-pyran-2-yl)oxy)-4*H*-chromen-8-yl acetate), and Compound **4**. (5,8-dihydroxy-2-(4-hydroxyphenyl)-7-methoxy-3-(((3*S*,4*S*,6*S*)-3,4,5-trihydroxy-6-methyltetrahydro-2*H*-pyran-2-yl)oxy)-4*H*-chromen-4-one). Additionally, this study provides a comprehensive micro-anatomical profile of the steam and leaf, along with an established TLC methodology for authenticating this plant species in commerce, preventing the distribution of unauthentic or adulterated samples. Further studies are necessary to comprehend, assess and validate the pharmacological properties of this plant.

## Figures and Tables

**Table 1:** Constituent composition of *A. pyrifolia* hexane extract.

No.	Compounds	Retention Time (RT)	Match Factor	Area (%)
1	Hentriacontane	38.0871	90.5	1.20
2	Nonacosane	38.4157	92.9	14.13
3	Heptacosane	38.4919	72.5	2.47
4	1,3-Benzenediol, 5-pentadecyl	47.0063	76.4	8.32
5	Campesterol	49.1682	92.3	4.31
6	n-Tetracosanol-1	54.7826	95.2	4.18
7	*γ*-sitosterol	55.7683	94.6	33.99
8	Stigmastanol	56.5302	81.3	1.82
9	*β*-amyrin	57.7541	74.4	1.00
10	Hexacosanal	58.5922	86.7	3.70
11	Lup-20(29)-en-3-one	59.0112	94.3	10.12
12	1-Hexacosanol	60.6303	95.3	6.65

**Table 2:** NMR Spectroscopic data for the isolated compounds from *A. pyrifolia* leaves.

Position	Compound 1 ^a^		Compound 2 ^a^			Compound 3 ^a^		Compound 4 ^b^	
	δ_C_, Type	δ_H_, (*J* in Hz)	δ_C_, Type	δ_H_, (*J* in Hz)	HMBC ^c^	δ_C_, Type	δ_H_, (*J* in Hz)	δ_C_, Type	δ_H_, (*J* in Hz)
2	158.72, C		159.12, C			159.63, C		159.63, C	
3	136.39, C		136.36, C			135.93, C		135.90, C	
4	179.70, C		179.59, C			180.12, C		180.05, C	
5	159.33, C		160.34, C			154.81, C		154.80, C	
6	96.31, CH_3_	6.51, s	96.27, CH_3_	6.46 s	8, 4a	96.05, CH_3_	6.45, s	96.05, CH_3_	6.51, s
7	160.40, C		158.63, C			155.03, C		154.99, C	
8	122.66, C		120.35, C			127.56, C		127.53,C	
8a	150.09, C		148.72, C			145.07, C		145.04, C	
4a	105.74, C		105.71, C			106.04, C		106.05, C	
1′	122.91, C		122.29, C			123.06, C		122,74, C	
2′	116.42, CH	6.92, d (2.1)	131.83, CH	7.69, d (8.9)	2, 4′	117.16, CH	7.44, d (2.1)	132.18, CH	7.87, m
3′	148.81, C		116.60, CH	6.93, d (8.9)	1′	146.32, C		116.43, CH	6.94, m
4′	146.51, C		161.79, CH			149.86, C		161.58, C	
5′	116.82, CH	7.30, d (8.4)	116.60, CH	6.93, d (8.9)	1′	116.29, CH	6.91, d (8.3)	116.43, CH	6.94, m
6′	120.42, CH	7.27, dd (8.4, 2.1)	131.83, CH	7.69, d (8.9)	2, 4′	123.21, CH	7.41, dd (8.3, 2.0)	132.18, CH	7.87, m
Rha-1	103.66, CH	5.36, d (1.8)	103.62, CH	5.38, d (1.7)	3, Rha-2	103.52, CH	5.37, d (1.6)	103.48, CH	5.39, d (1.7)
Rha-2	71.89, CH	4.23, dd (3.4, 1.8)	71.90, CH	4.24, dd (3.4, 1.8)	Rha-4	71.91, CH	4.24, m	71.92, CH	4.23, dd (3.4, 1.7)
Rha-3	72.09, CH	3.76, dd (9.5, 3.5)	71.90, CH	3.73, dd (9.2, 3.4)	Rha-5	72.04, CH	3.77, dd (9.5, 3.3)	72.05, CH	3.73, dd (9.0, 3.3)
Rha-4	72.09, CH	3.45, m	72.08, CH	3.35, m		73.26, CH	3.35 ^d^	72.12, CH	3.36, m
Rha-5	73.22, CH	3.34, t (9.5)	73.16, CH	3.35, m	Rha-4	72.11, CH	3.45, dq (9.6, 6.1)	73.20, CH	3.36, m
Rha-6	17.56, CH	0.95, d (6.3)	17.63, CH_3_	0.93, d (6.0)	Rha-5	17.65, CH_3_	0.95, d (6.2)	17.66, CH_3_	0.93, d (5.8)
7-OMe	57.18, CH_3_	3.89, s	57.15, CH_3_	3.87, s	7	56.95, CH_3_	3.93, s	56.94, CH_3_	3.96, s
8-acetyl (C=O)	170.36, C		170.26, C						
8-acetyl (CH_3_)	20.06, CH_3_	2.33, s	20.04, CH_3_	2.28, s	8-acetyl (C=O)				

In methanol-d4. ^a^ 500 MHz ^b^ 400 MHz ^c^ HMBC correlation H→C. ^d^ Unclear signal pattern due to overlapping.

## Data Availability

Data is contained within the article and Supplementary Materials.

## Supplementary Materials

The following supporting information can be downloaded at: https://www.mdpi.com/article/10.3390/molecules29040833/s1, Figure S1. GC-MS spectra of hexane extract of *A. pyrifolia* leaves; Figure S2. ^1^H and ^13^C NMR of Compound **1**; Figure S3. HSQC and HMBC NMR correlations of Compound **1**; Figure S4. ^1^H and ^13^C NMR of Compound **2**; Figure S5. HSQC and HMBC correlations of Compound **2**; Figure S6. ^1^H and ^13^C NMR correlations of Compound **3**; Figure S7. HSQC and HMBC NMR correlations of Compound **3**; Figure S8. ^1^H and ^13^C NMR correlations of Compound **4**; Figure S9. HSQC and HMBC NMR correlations of Compound **4**; Figure S10. HR-ESI-MS [M + H]^+^ data of Compound **1**; Figure S11. HR-ESI-MS [M + H]^+^ data of Compound **2**; Figure S12. HR-ESI-MS [M + H]^−^ data of Compound **3**; Figure S13. HR-ESI-MS [M + H]^−^ data of Compound **4**.
