# Supplementary material for: Phytochemical and Micro-Morphological Characterization of Atraphaxis pyrifolia Bunge Growing in the Republic of Kazakhstan
Source: Molecules. 2024 Feb 13;29(4):833. doi: 10.3390/molecules29040833 (PMC10891614; doi:10.3390/molecules29040833)
Supplement: Supplementary file 1 [file molecules-29-00833-s001.zip › molecules-2846471-supplementary.pdf]

*Supplementary material*

# Phytochemical and Micro-Morphological Characterization of *Atraphaxis pyrifolia* Bunge Growing in the Republic of Kazakhstan

**Alima Abilkassymova 1,2,3, Raushan Kozykeyeva 3,4 , Jennyfer Andrea Aldana-Mejía 3, Sebastian John Adams 3 , Ubaidilla Datkhayev 2, Aknur Turgumbayeva 1,5, Kulpan Orynbassarova 4, Seethapathy G. Saroja 3 , Ikhlas A. Khan 3,6 and Samir A. Ross 2,3,6,***

[1] Higher School of Medicine, Al-Farabi Kazakh National University, Almaty 050040, Kazakhstan; abilkasymova_a@mail.ru (A.A.); turgumbayeva.aknur@med-kaznu.com (A.T.)
[2] School of Pharmacy, Asfendiyarov Kazakh National Medical University, Almaty 050012, Kazakhstan; u.datkhayev@gmail.com
[3] National Center for Natural Products Research, School of Pharmacy, The University of Mississippi, Oxford, MS 38677, USA; nar_rau@mail.ru (R.K.); jaaldana@olemiss.edu (J.A.A.-M.); jasabest@olemiss.edu (S.J.A.); seethapathy.gs@gmail.com (S.G.S.); ikhan@olemiss.edu (I.A.K.)
[4] Department of Pharmacognosy, Faculty of Pharmacy, South Kazakhstan Medical Academy, Shymkent 160019, Kazakhstan; kulpan_ok@mail.ru
[5] School of Life Sciences, University of Westminster, London W1W 6UW, UK
[6] Department of Biomolecular Sciences, Division of Pharmacognosy, School of Pharmacy, University of Mississippi, Oxford, MS 38677, USA

## Table of contents

**Figure S 1.** GC-MS spectra of hexane extract of *A. pyrifolia* leaves. ..........3
**Figure S 2.** $^1$H and $^{13}$C NMR (MeOD; 500 MHz) of Compound 1 (8-*O*-acetyl-7-*O*-methyl-3-*O*-α-L-rhamnopyranosylgossypetin). ..........4
**Figure S 3.** HSQC and HMBC NMR correlations (MeOD; 500 MHz) of Compound 1 (8-*O*-acetyl-7-*O*-methyl-3-*O*-α-L-rhamnopyranosylgossypetin). ..........5
**Figure S 4.** $^1$H and $^{13}$C NMR (MeOD; 500 MHz) of Compound **2** (5-hydroxy-2-(4-hydroxyphenyl)-7-methoxy-4-oxo-3-(((3S,4S,6S)-3,4,5-trihydroxy-6-methyltetrahydro-2H-pyran-2-yl)oxy)-4H-chromen-8-yl acetate)..........6
**Figure S 5.** HSQC and HMBC correlations (MeOD; 500 MHz) of Compound **2** (5-hydroxy-2-(4-hydroxyphenyl)-7-methoxy-4-oxo-3-(((3S,4S,6S)-3,4,5-trihydroxy-6-methyltetrahydro-2H-pyran-2-yl)oxy)-4H-chromen-8-yl acetate). ..........7
**Figure S 6.** $^1$H and $^{13}$C NMR correlations (MeOD; 500 MHz) of Compound **3** (7-*O*-methyl-3-*O*-α-L-rhamnopyranosylgossypetin). ..........8
**Figure S 7.** HSQC and HMBC NMR correlations (MeOD; 500 MHz) of Compound **3** (7-*O*-methyl-3-*O*-α-L-rhamnopyranosylgossypetin). ..........9
**Figure S 8.** $^1$H and $^{13}$C NMR correlations (MeOD; 400 MHz) of Compound **4** (5,8-dihydroxy-2-(4-hydroxyphenyl)-7-methoxy-3-(((3S,4S,6S)-3,4,5-trihydroxy-6-methyltetrahydro-2H-pyran-2-yl)oxy)-4H-chromen-4-one). ..........10

**Figure S 9.** HSQC and HMBC NMR correlations (MeOD; 400 MHz) of Compound **4** (5,8-dihydroxy-2-(4-hydroxyphenyl)-7-methoxy-3-(((3S,4S,6S)-3,4,5-trihydroxy-6-methyltetrahydro-2H-pyran-2-yl)oxy)-4H-chromen-4-one). ....11
**Figure S 10.** HR-ESI-MS $[M + H]^+$ data of Compound **1**. ....12
**Figure S 11.** HR-ESI-MS $[M^+H]^+$ data of Compound **2**. ....13
**Figure S 12.** HR-ESI-MS $[M^+H]^-$ data of Compound **3**. ....14
**Figure S 13.** HR-ESI-MS $[M^+H]^-$ data of Compound **4**. ....15

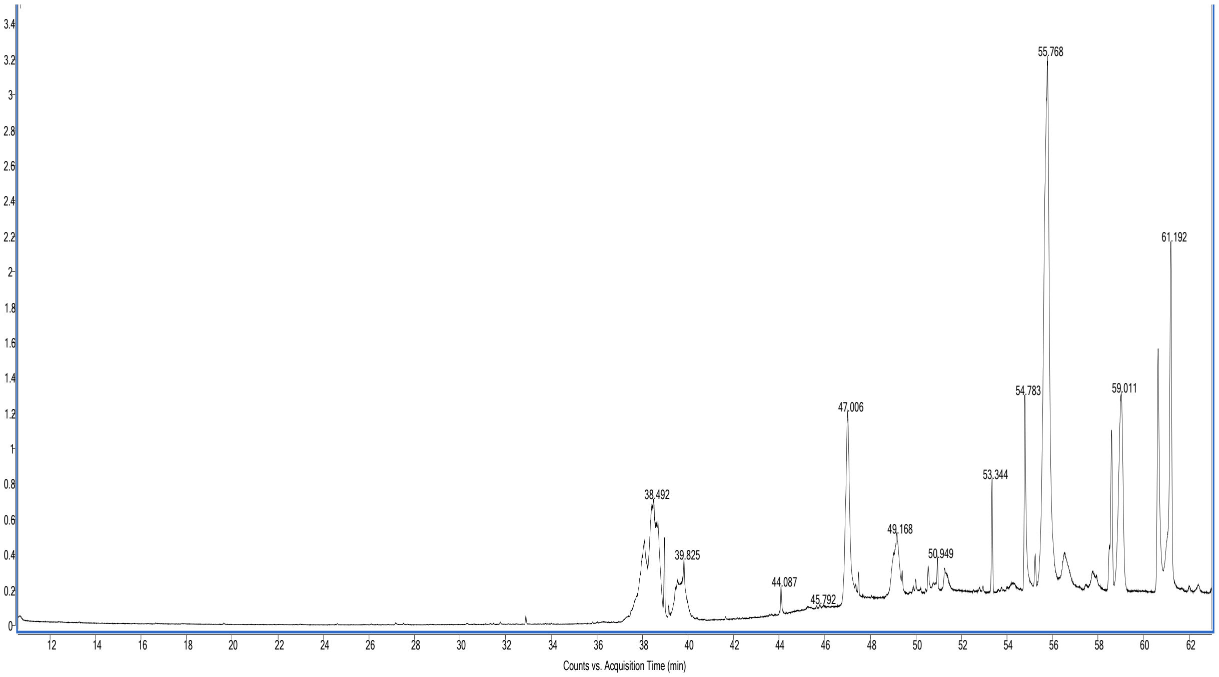


**Figure S 1.** GC-MS spectra of hexane extract of *A. pyrifolia* leaves.

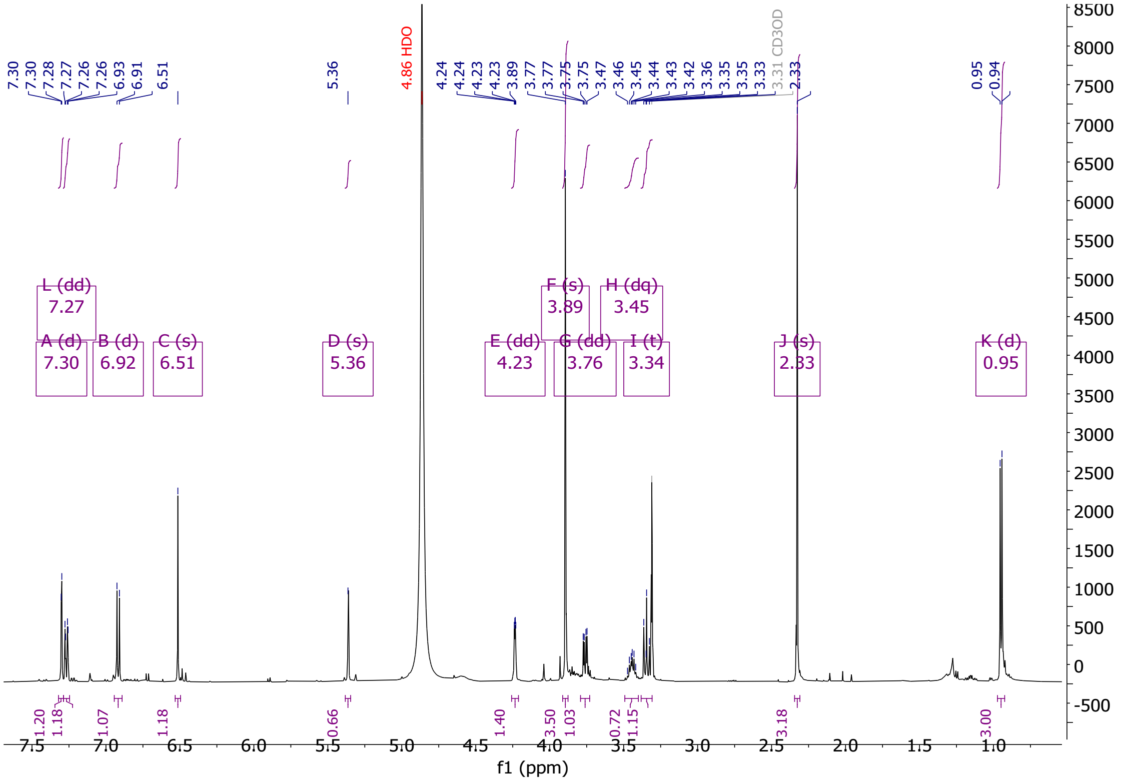


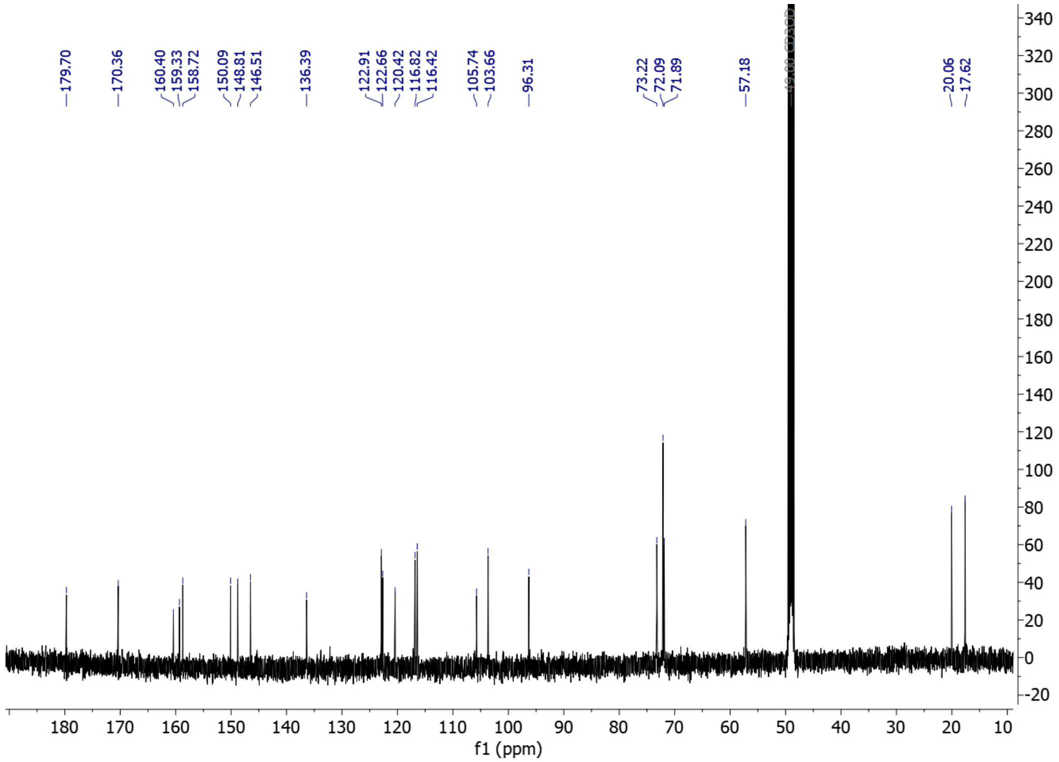


**Figure S 2.** $^{1}H$ and $^{13}C$ NMR (MeOD; 500 MHz) of Compound **1** (8-*O*-acetyl-7-*O*-methyl-3-*O*-α-L-rhamnopyranosylgossypetin).

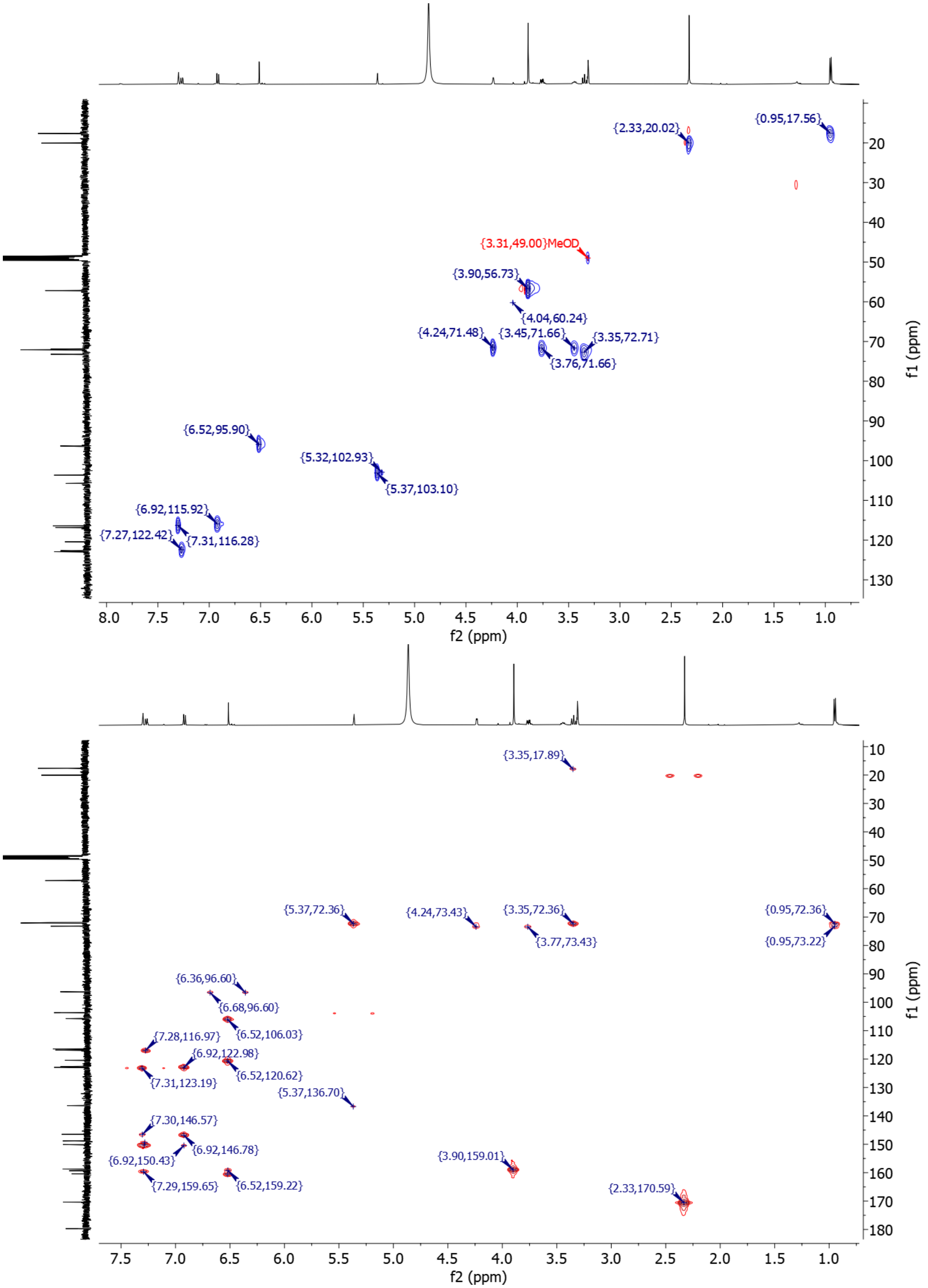


**Figure S 3.** HSQC and HMBC NMR correlations (MeOD; 500 MHz) of Compound **1** (8-*O*-acetyl-7-*O*-methyl-3-*O*-$\alpha$-L-rhamnopyranosylgossypetin).

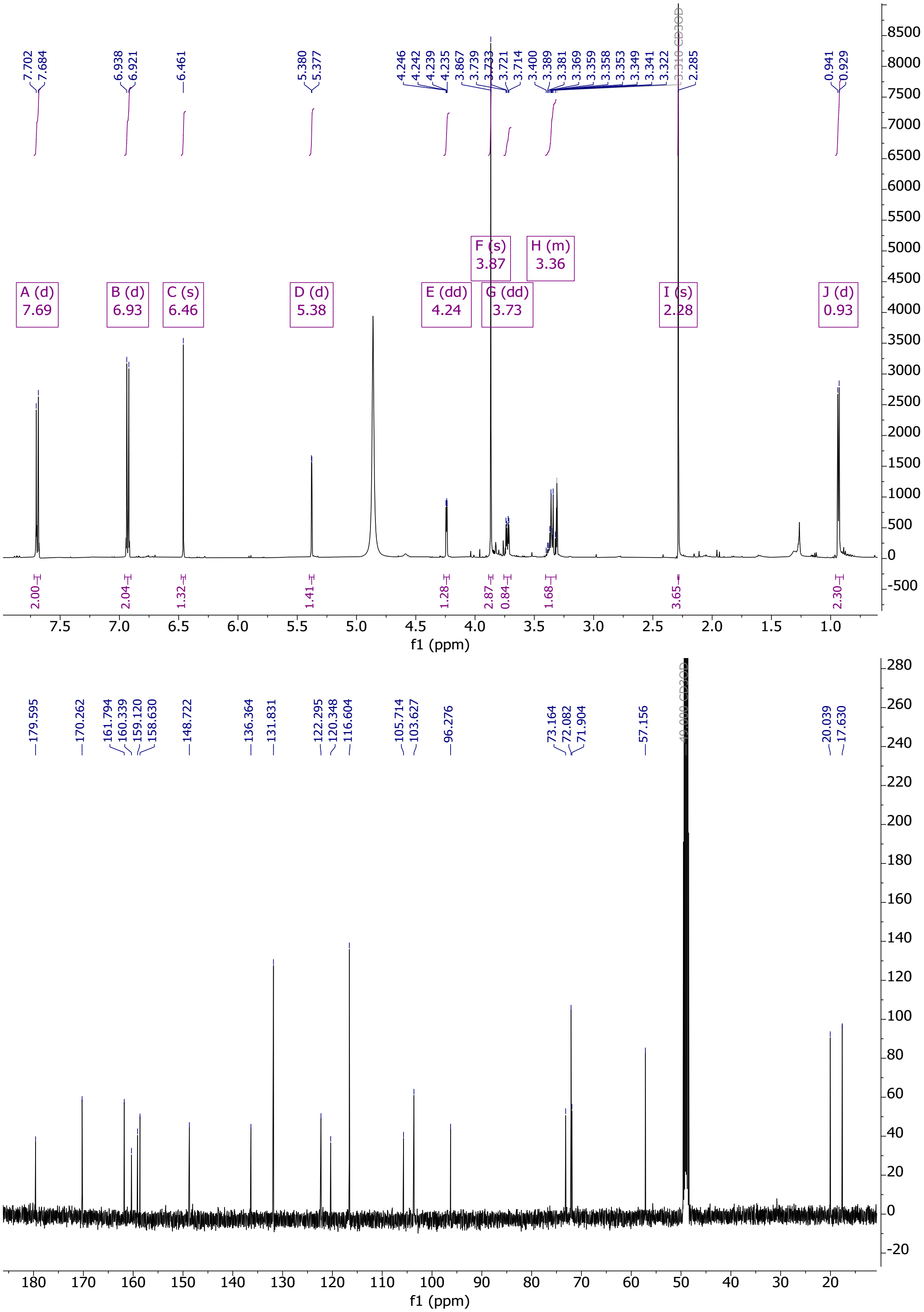


**Figure S 4.** $^{1}H$ and $^{13}C$ NMR (MeOD; 500 MHz) of Compound **2** (5-hydroxy-2-(4-hydroxyphenyl)-7-methoxy-4-oxo-3-(((3S,4S,6S)-3,4,5-trihydroxy-6-methyltetrahydro-2H-pyran-2-yl)oxy)-4H-chromen-8-yl acetate).

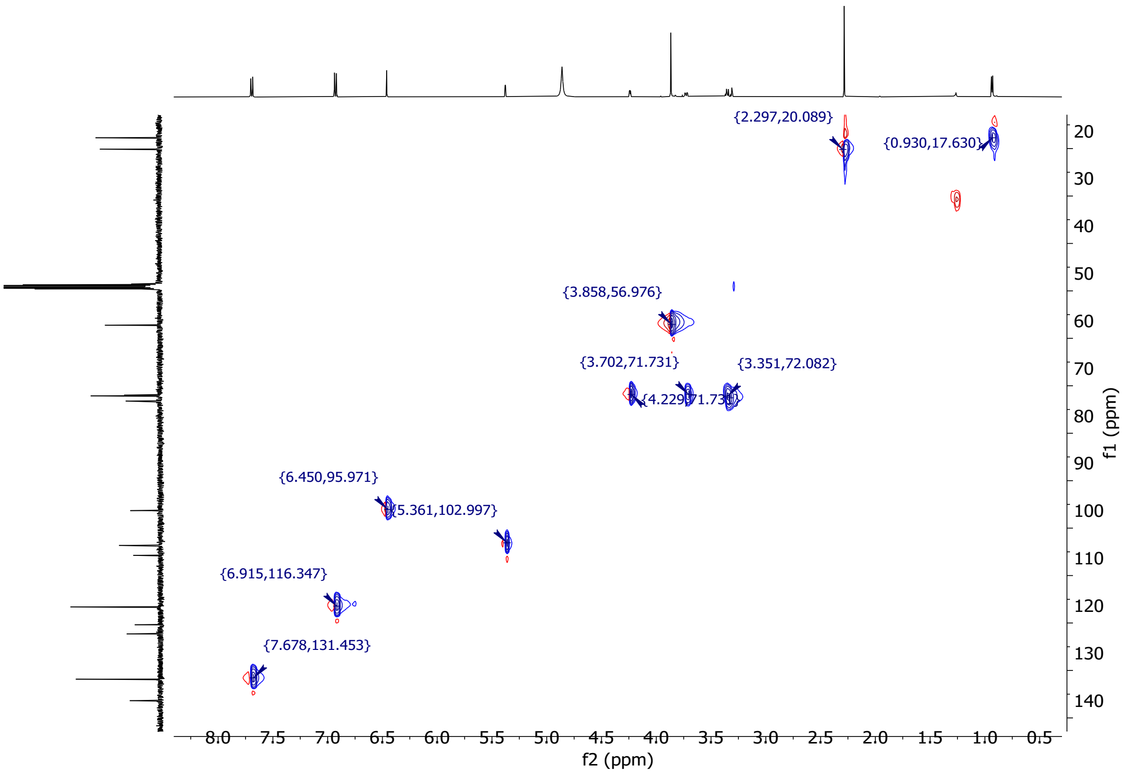


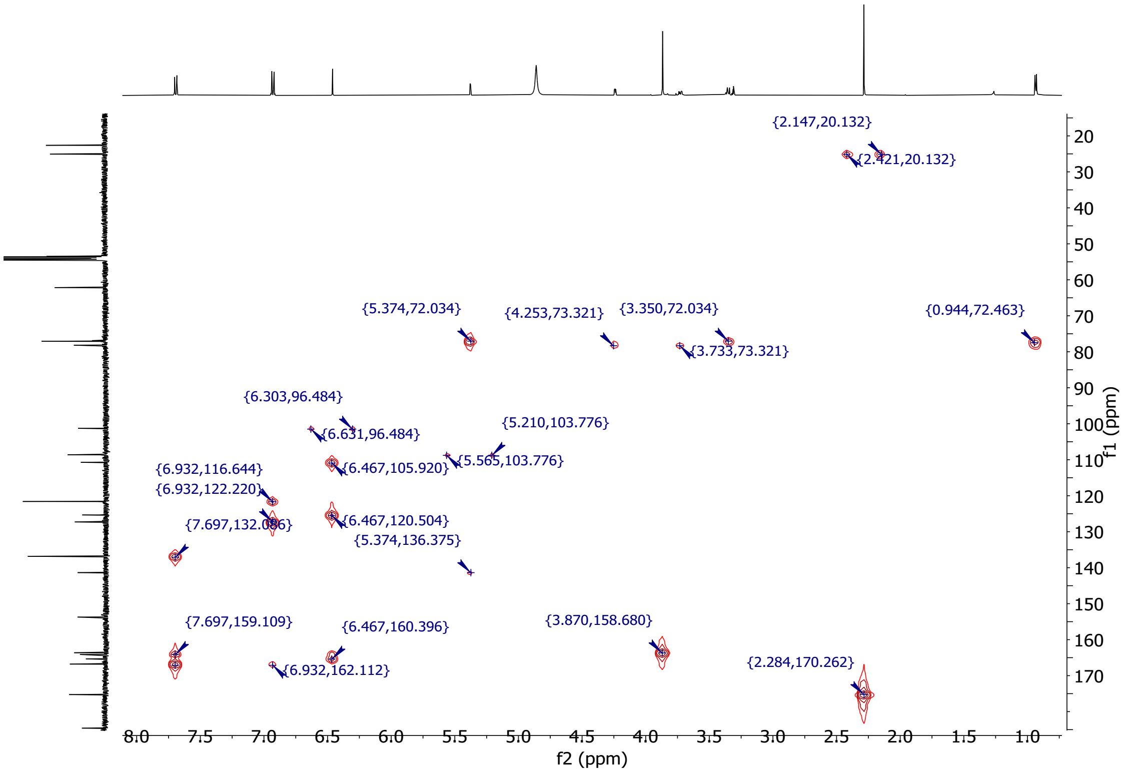


**Figure S 5.** HSQC and HMBC correlations (MeOD; 500 MHz) of Compound **2** (5-hydroxy-2-(4-hydroxyphenyl)-7-methoxy-4-oxo-3-(((3S,4S,6S)-3,4,5-trihydroxy-6-methyltetrahydro-2H-pyran-2-yl)oxy)-4H-chromen-8-yl acetate).

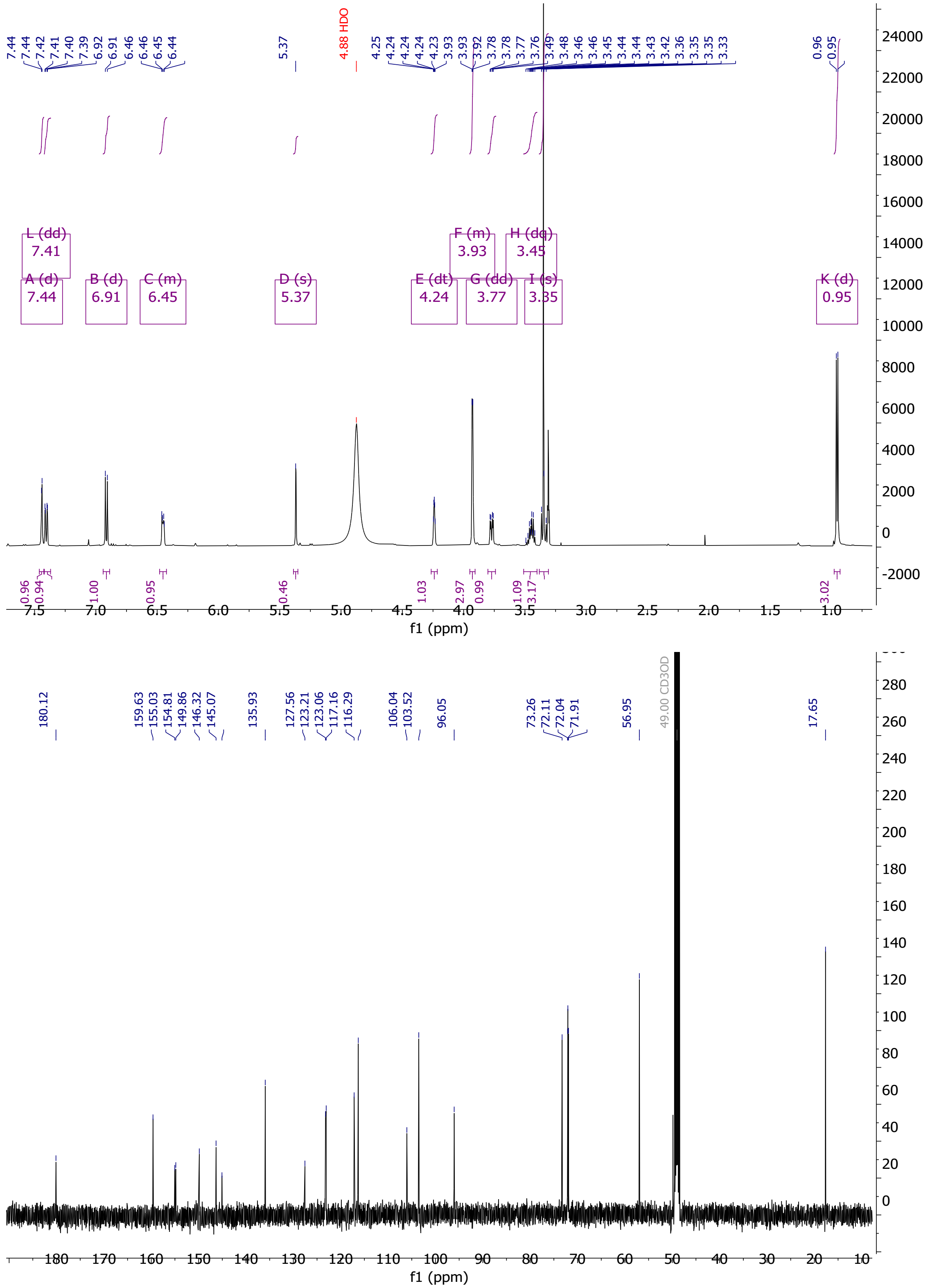


**Figure S 6.** $^{1}$H and $^{13}$C NMR correlations (MeOD; 500 MHz) of Compound **3** (7-*O*-methyl-3-*O*-α-L-rhamnopyranosylgossypetin).

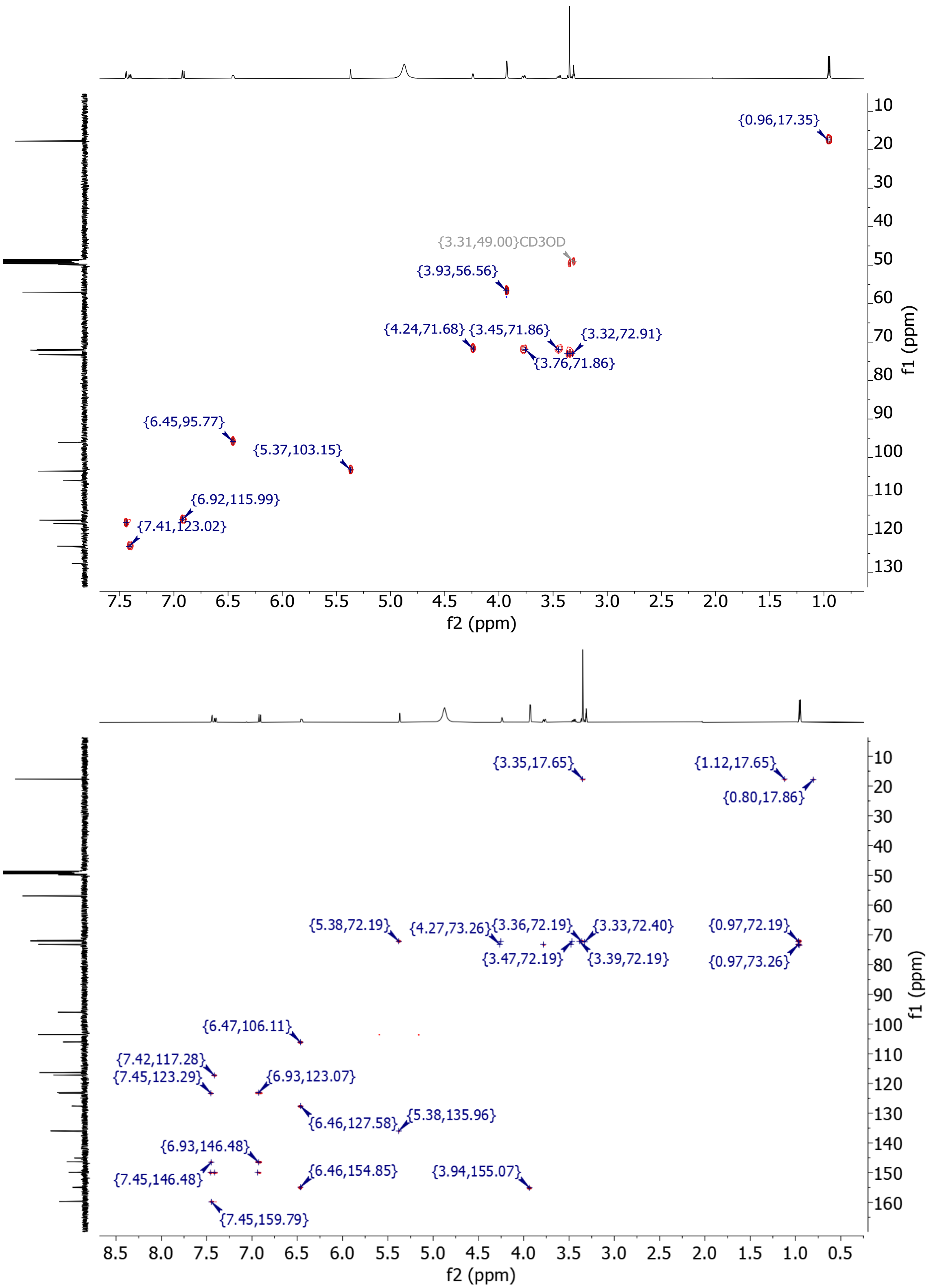


**Figure S 7.** HSQC and HMBC NMR correlations (MeOD; 500 MHz) of Compound **3** (7-*O*-methyl-3-*O*-α-L-rhamnopyranosylgossypetin).

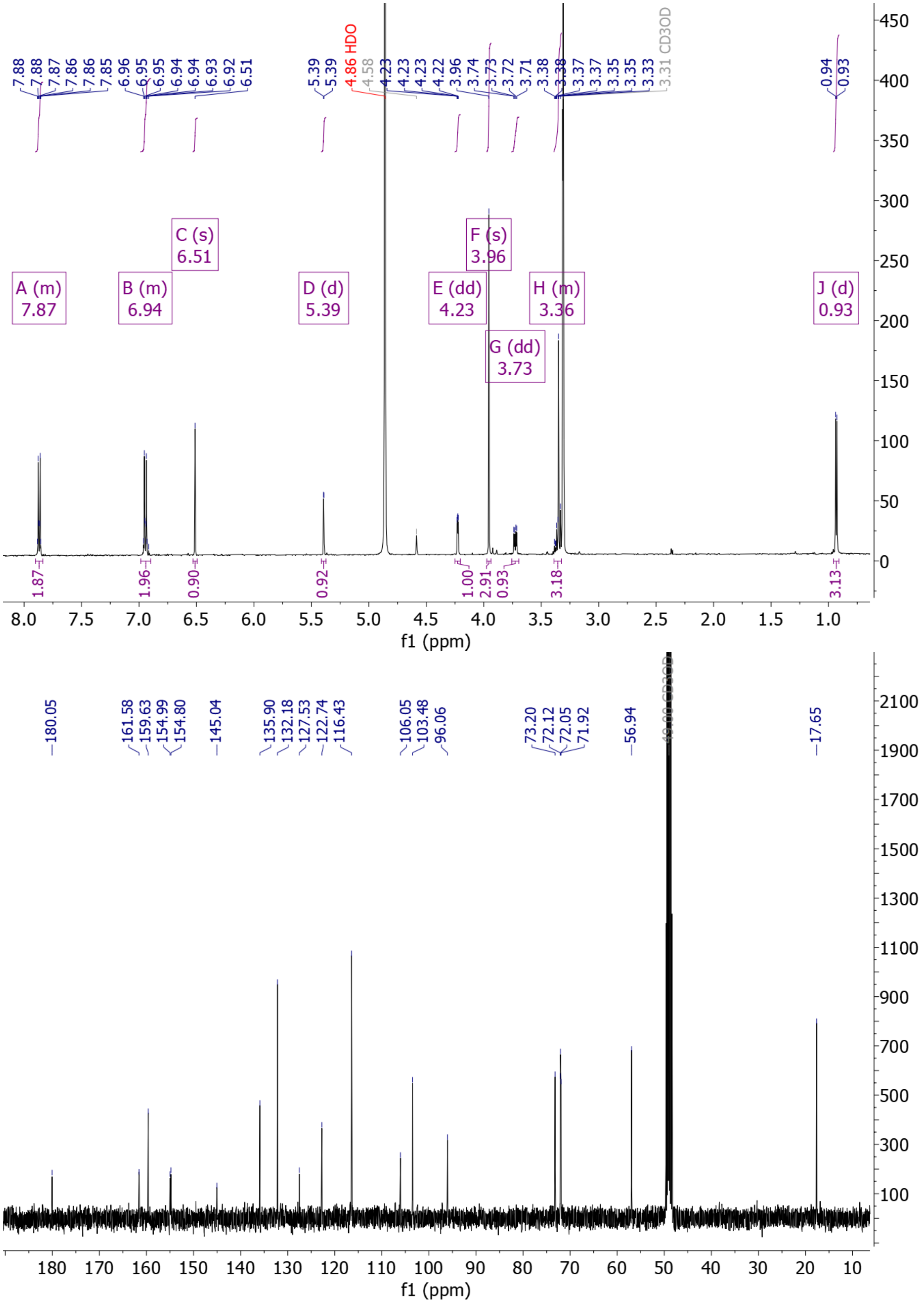


**Figure S 8.** $^{1}H$ and $^{13}C$ NMR correlations (MeOD; 400 MHz) of Compound **4** (5,8-dihydroxy-2-(4-hydroxyphenyl)-7-methoxy-3-(((3S,4S,6S)-3,4,5-trihydroxy-6-methyltetrahydro-2H-pyran-2-yl)oxy)-4H-chromen-4-one).

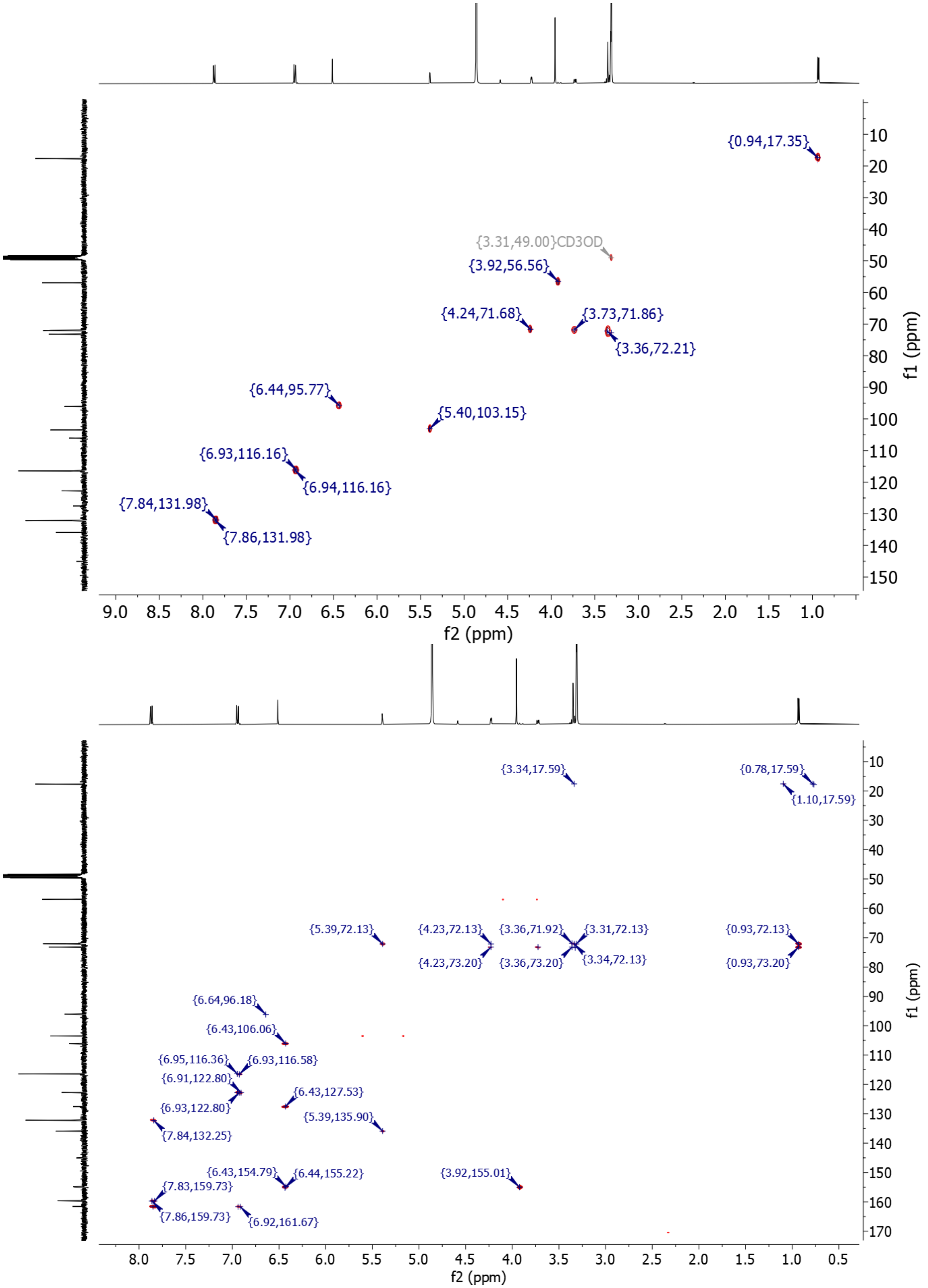


**Figure S 9.** HSQC and HMBC NMR correlations (MeOD; 400 MHz) of Compound **4** (5,8-dihydroxy-2-(4-hydroxyphenyl)-7-methoxy-3-(((3S,4S,6S)-3,4,5-trihydroxy-6-methyltetrahydro-2H-pyran-2-yl)oxy)-4H-chromen-4-one).

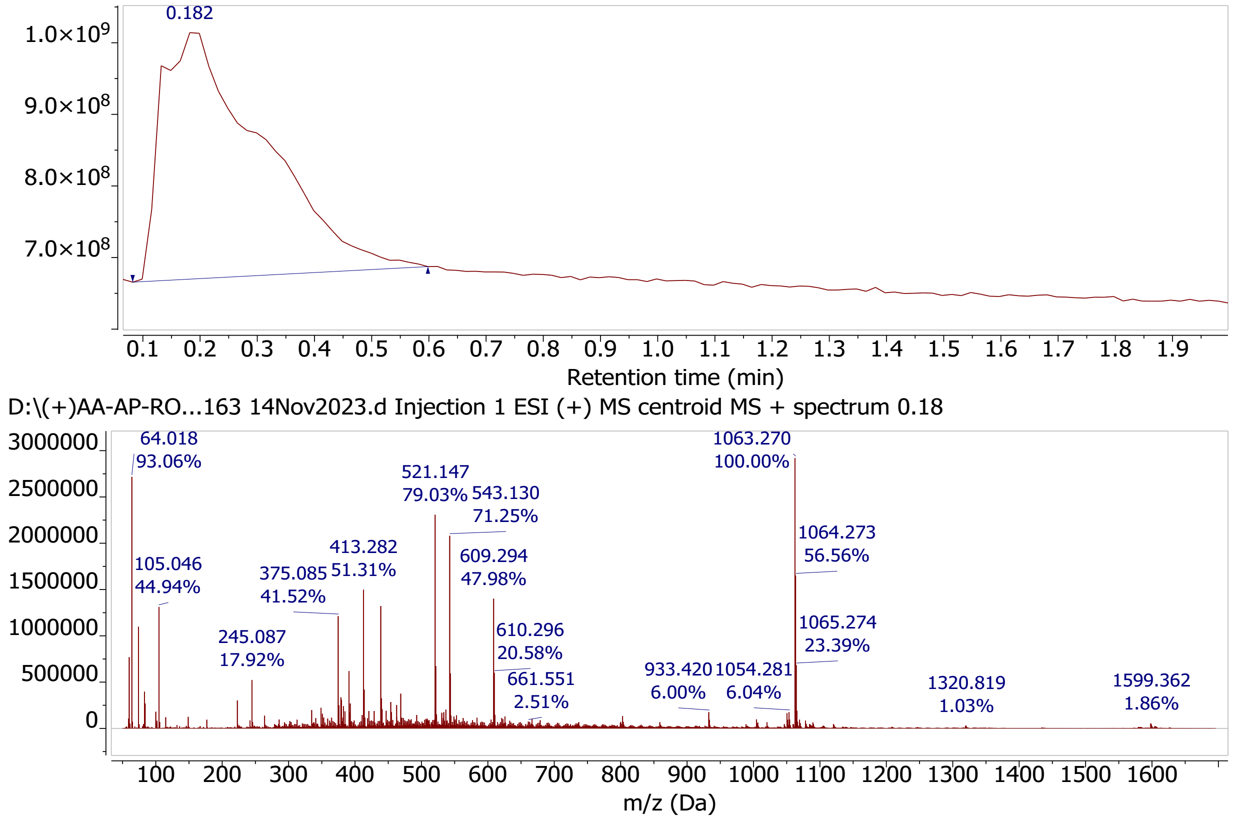


**Figure S 10.** HR-ESI-MS $[M + H]^+$ data of Compound **1**.

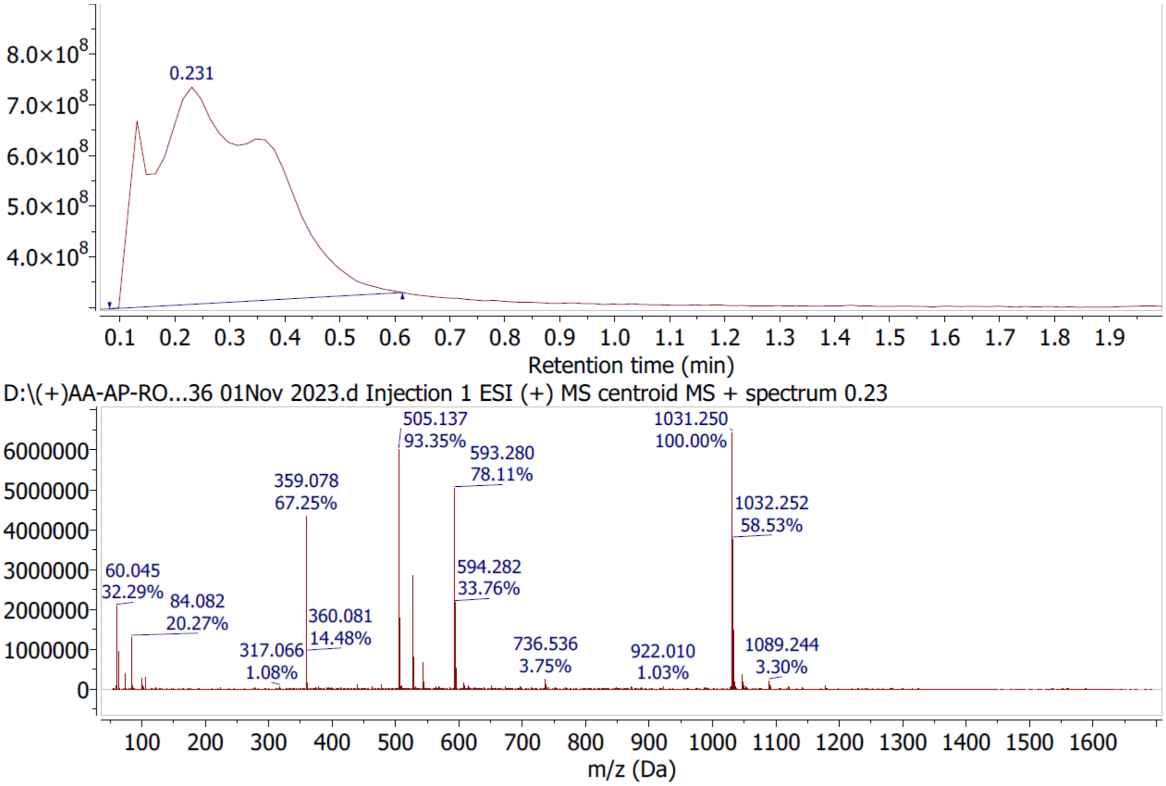


**Figure S 11.** HR-ESI-MS $[M^{+}H]^{+}$ data of Compound **2**.

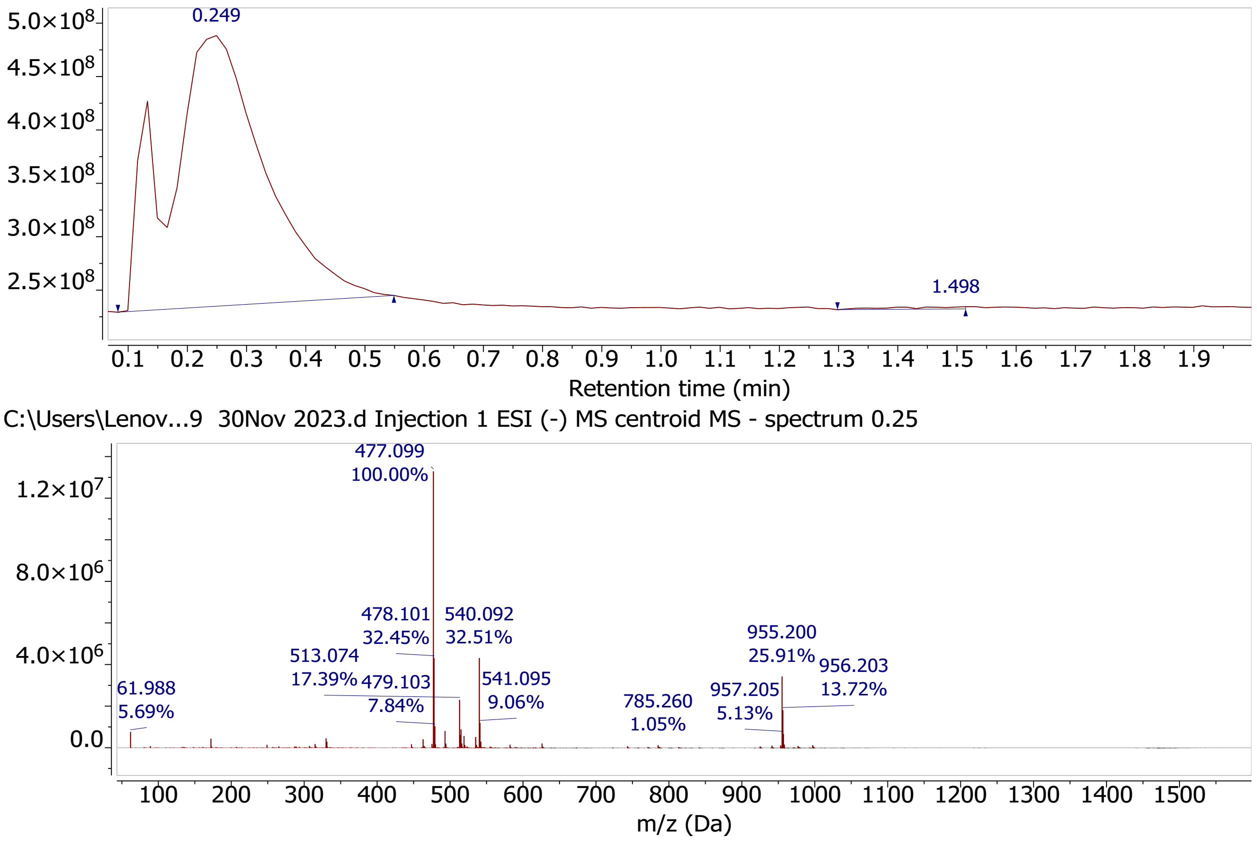


**Figure S 12.** HR-ESI-MS $[M^{+}H]^{-}$ data of Compound **3**.

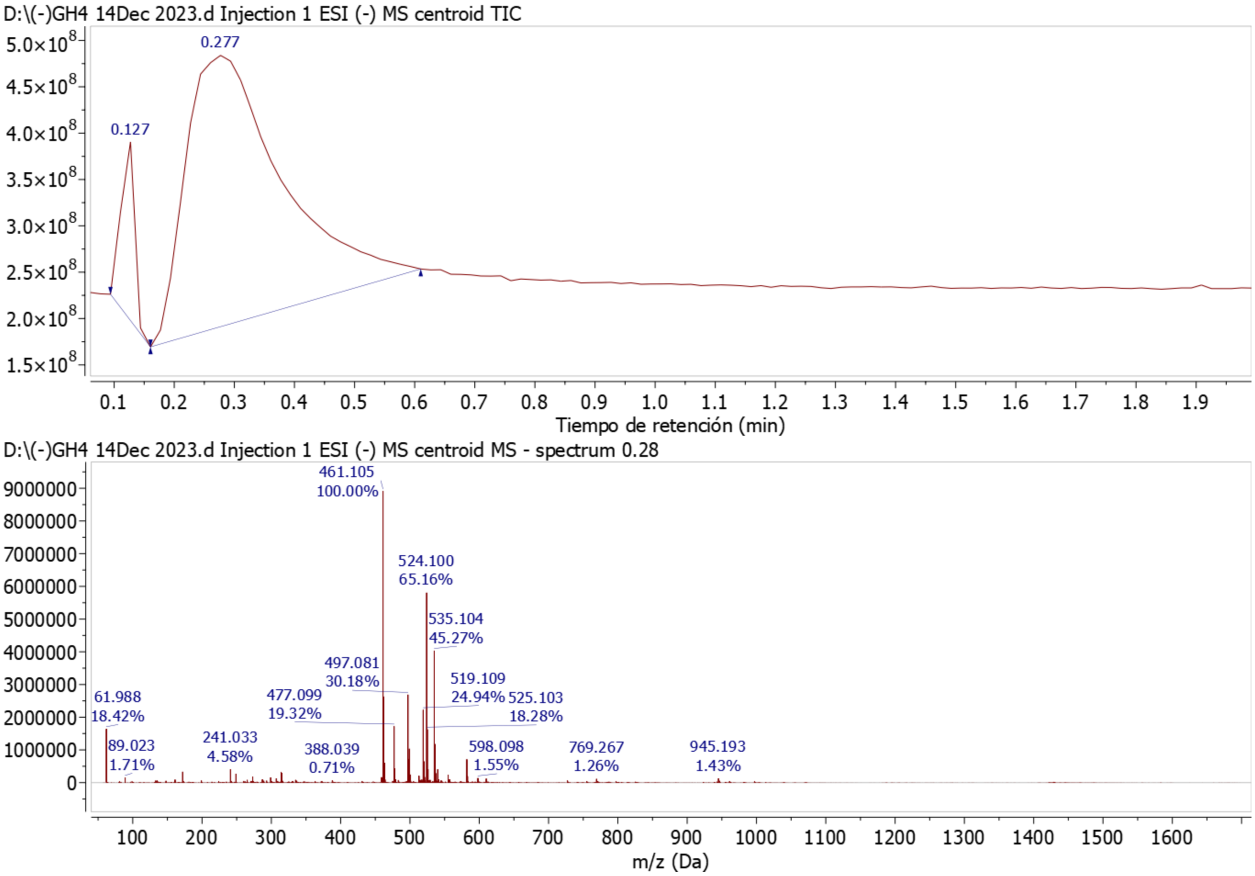


**Figure S 13.** HR-ESI-MS [M+H]- data of Compound **4**.
